# Innovative Biosensor Platforms for the Detection of Metformin in Diabetes Management

**DOI:** 10.1155/ianc/8261007

**Published:** 2026-05-29

**Authors:** Sajjad Jafarzadeh, Elham Shaterian, Samira Jafarisis, Mahya Mohammadi, Hossein Azizian, Shahla Shiri, Ahmad Mobed

**Affiliations:** ^1^ Department of Stem Cells and Developmental Biology, Cell Sciences Center, Royan Institute for Stem Cell Biology and Technology, ACECR, Tehran, Iran, acecr.ac.ir; ^2^ Department of Medicinal Chemistry, Faculty of Pharmacy, Tabriz University of Medical Sciences, Tabriz, Iran, tbzmed.ac.ir; ^3^ Cardiovascular Fellowship, Jacob School of Medicine, University of Buffalo, Buffalo, New York, USA, buffalo.edu; ^4^ Student Research Committee, School of Medicine, Shahid Beheshti University of Medical Sciences, Tehran, Iran, sbmu.ac.ir; ^5^ Yazd Neuroendocrine Research Center, School of Medicine, Shahid Sadoughi University of Medical Sciences, Yazd, Iran, ssu.ac.ir; ^6^ Biosensor Sciences and Technologies Research Center, Ardabil University of Medical Sciences, Ardabil, Iran, arums.ac.ir

**Keywords:** biosensors, clinical and pharmaceutical application, metformin, type 2 diabetes

## Abstract

Metformin, a widely prescribed antihyperglycemic agent, plays a crucial role in the management of Type 2 diabetes by reducing hepatic glucose production and increasing insulin sensitivity. Its effectiveness in glucose regulation has made it a cornerstone in diabetes care, necessitating precise monitoring of drug levels to optimize therapeutic outcomes and minimize potential adverse effects. This review explores contemporary biosensor technologies designed for the sensitive detection of metformin, emphasizing their significance in clinical and pharmaceutical settings. We analyze various biosensor platforms, including electrochemical, optical, and piezoelectric systems, highlighting their principles, advantages, and challenges. Additionally, we discuss the integration of nanomaterials to enhance detection sensitivity and specificity. Given the rising prevalence of diabetes globally, the development of innovative biosensing strategies for metformin detection is paramount in ensuring effective patient management and improving treatment adherence. The insights gained from this review aim to propel further research and development in this vital area of biomedical engineering.

## 1. Introduction

Diabetes mellitus (DM), particularly Type 2 diabetes mellitus (T2DM), poses an increasing threat to global public health, contributing to significant rates of cardiovascular disease, limb‐related morbidities, and mortality [1, 2]. Current estimates indicate that in 2021, approximately 4.5% of adults aged 20–79 in sub‐Saharan Africa were diagnosed with diabetes, a figure projected to rise dramatically by 129% to 55 million individuals by 2045 [1, 2]. This alarming trend underscores the urgent need for effective management strategies for T2DM, especially considering that over 54% of diabetes cases in this region remain undiagnosed. In conjunction with this rise, healthcare expenditures related to diabetes are ballooning, anticipating a jump to USD 6 billion by 2045 [3, 4]. Globally, the statistics are equally striking, with an estimated 529 million individuals affected by T2DM as of 2021, the majority being elderly adults [3, 4]. This widespread prevalence is not just a health crisis but also leads to numerous complications, including peripheral artery disease (PAD), which affects approximately 14% of those diagnosed [5, 6]. The atherosclerotic changes associated with PAD lead to severely diminished functional capacity and heightened mortality. Within this context, metformin emerges as a pivotal player in T2DM management. As a widely prescribed antihyperglycemic agent, it effectively reduces hepatic glucose production and enhances insulin sensitivity [5, 6]. Beyond glycemic control, recent studies have suggested that metformin could have additional benefits, including a significant reduction in all‐cause and cardiovascular mortality among T2DM patients [7, 8]. However, its effectiveness can be influenced by factors such as drug adherence and precise monitoring of blood levels, which necessitate advanced detection methodologies [7, 8]. Recent progresses in biosensor technology have enabled sensitive, selective, and potential point‐of‐care detection of metformin using electrochemical, optical, and hybrid platforms, often enhanced by nanomaterials such as metal oxides, graphene derivatives, carbon nanotubes (CNTs), and molecularly imprinted polymers. These advances address limitations of conventional analytical methods (e.g., HPLC, spectroscopy), present faster response times, lower detection limits, and improved performance in complex biological matrices such as plasma, urine, or interstitial fluid [9]. Emerging wearable and microneedle‐based systems further support real‐time therapeutic drug monitoring (TDM) and personalized diabetes management. However, challenges persist in long‐term stability, anti‐interference capability, scalability, and clinical translation. This review critically evaluates these biosensor platforms, their underlying principles, nanomaterial contributions, common bottlenecks, and future directions to guide research toward practical, effective metformin monitoring tools.

## 2. Metformin

**FIGURE 1:**
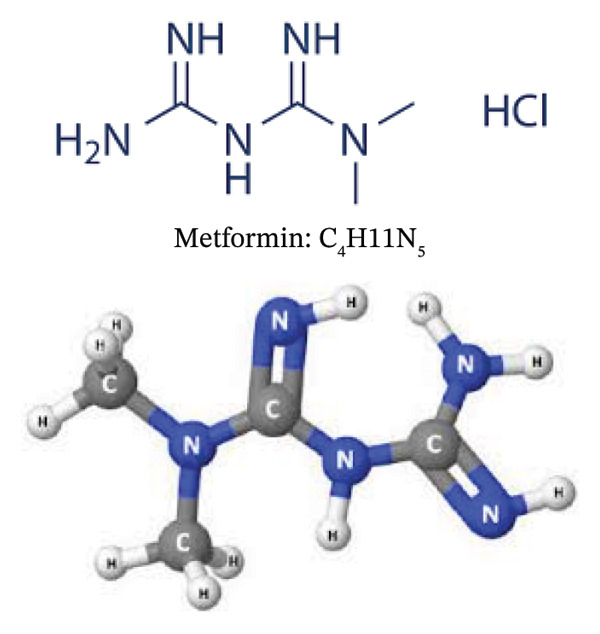
Chemical structure of metformin: metformin hydrochloride (1,1‐dimethylbiguanide hydrochloride) (C_4_H11N_5_).

The history of metformin can be traced back to the herbal use of *Galega officinalis*, commonly known as goat’s rue or French lilac [10, 11]. This plant was documented in the 1700s as a traditional remedy for symptoms associated with diabetes, namely, excessive thirst and frequent urination [10, 11]. By the mid‐1800s, it was discovered that G. officinalis contained guanidine, a compound later recognized for its ability to lower blood glucose levels [10, 11]. The chemical structure of metformin (metformin hydrochloride (1,1‐dimethylbiguanide hydrochloride)) (C_4_H11N_5_) is presented in Figure 1.

In 1918, guanidine itself was shown to effectively reduce blood sugar in animal models, marking a pivotal moment in diabetes treatment [12]. In the 1920s, several monoguanidine derivatives were synthesized, notably *galegine* and *synthalin*, with the aim of managing diabetes [12]. However, these compounds were met with limitations in efficacy and safety concerns due to their toxic effects. Concurrently, the increasing availability of insulin rendered these early treatments less relevant, leading to their decline in use during the 1930s and early 1940s [13, 14]. Metformin, derived from compound structures related to guanidine, has become a foundational therapy for Type 2 diabetes since its introduction in the 1950s [13, 14]. Its effectiveness in managing blood glucose levels has been widely recognized, but its applications extend beyond diabetes. Current clinical uses include treatment for polycystic ovarian syndrome, management of diabetes during pregnancy and gestational diabetes, prevention of Type 2 diabetes in individuals with prediabetes, and as an adjunct therapy in Type 1 diabetes. Research indicates that many of metformin’s beneficial effects may stem from modest impacts on weight loss and improved insulin sensitivity. Additionally, the drug exhibits multiple mechanisms of action, contributing to its utility in various metabolic conditions. Importantly, as metformin has been in clinical use for nearly 70 years, much of the supporting evidence for its applications in these diverse contexts is derived from studies conducted before the establishment of modern evidence‐based medicine principles. This historical context underscores the drug’s longstanding role in diabetes management and emerging therapeutic areas. Upon oral intake, 50% of metformin is absorbed through passive diffusion, while the remainder is taken up via facilitated diffusion through PMAT and organic cation transporter 1 (OCT1) transporters in the intestinal cells. After absorption, metformin exits the enterocytes through OCT1 and enters the liver via the portal vein, achieving concentrations between 40 and 70 μM. It then enters liver cells through OCT1 and organic cation transporter 3 (OCT3), where it inhibits gluconeogenesis [15].

**FIGURE 2:**
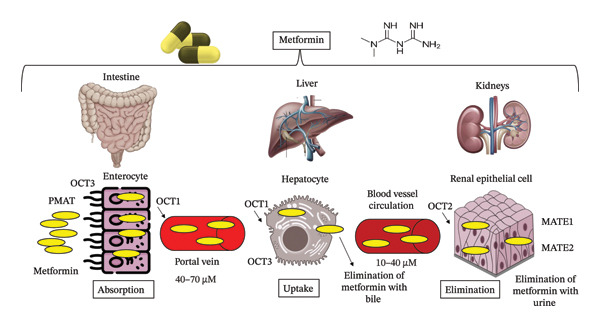
Schematic representation of metformin’s pharmacokinetics in the human body. After oral administration, metformin is absorbed in the intestine and transported to the liver where it inhibits gluconeogenesis. It is not metabolized but is excreted unchanged through the bile and kidneys, highlighting key transporters involved in its absorption and elimination: PMAT, OCT1, OCT2, OCT3, MATE1, and MATE2.

As illustrated in Figure 2, the liver does not metabolize the drug; instead, multidrug and toxin extrusion protein 1 (MATE1), found in hepatocytes, helps eliminate the unmetabolized drug either through bile or by transporting it into the bloodstream for renal excretion. Next, metformin enters renal epithelial cells via OCT2. It is then secreted unchanged by renal MATE1 and multidrug and toxin extrusion protein 2 (MATE2) and is ultimately eliminated in urine [15].

## 3. Metformin Detection Methods and Their Importance

Metformin, a widely prescribed medication, is primarily used in the management of Type 2 diabetes [16]. Its importance extends beyond pharmacology, into areas such as forensics, food safety, and environmental monitoring [16]. Accurate detection methods are crucial for ensuring patient safety, regulatory compliance, and environmental health [17]. Accurate detection enables healthcare providers to monitor drug levels, ensuring therapeutic efficacy and avoiding potential toxicity [17]. It also plays a crucial role in patient safety by helping identify cases of overdose or renal impairment, where metformin clearance might be compromised [18]. In forensic applications, metformin detection can provide critical insights into a patient’s history and treatment needs in cases of suspected poisoning or drug abuse [19]. It may also be relevant in legal investigations involving illegal drug use or noncompliance with prescribed therapy [20]. For environmental monitoring, metformin can persist in the environment, making its detection in water supplies essential for indicating pharmaceutical contamination [21]. This prompts necessary interventions to protect public health. Additionally, understanding how pharmaceutical pollutants affect aquatic life and ecosystems is vital for environmental conservation efforts [21]. Regulatory compliance is another crucial aspect, particularly in food safety. Detecting metformin in livestock feeds ensures compliance with food safety standards and regulations [22]. Overall, methods for detecting metformin are crucial across multiple fields, ensuring the safety and efficacy of this important medication while also safeguarding public health and the environment. Several methods are employed to detect metformin in various samples, including biological fluids, food products, and environmental samples. High‐performance liquid chromatography (HPLC) utilizes a liquid chromatography technique to separate and quantify metformin [23]. This method is highly accurate and reliable, allowing for the analysis of multiple samples simultaneously. However, it requires expensive equipment and specialized expertise [23]. Liquid chromatography‐mass spectrometry (LC‐MS) combines liquid chromatography with mass spectrometry, enabling precise quantification and identification of metformin [24]. This method is very sensitive and can detect low concentrations, but it comes with high costs and technical complexity [24]. Gas chromatography (GC) involves vaporizing a sample and separating it in a gas phase, making it suitable for volatile components. However, it is not appropriate for nonvolatile compounds such as metformin unless derivatization is performed [25]. Enzyme‐linked immunosorbent assay (ELISA) is a biochemical assay that utilizes antibodies to detect metformin’s presence [26]. This method is relatively quick and easy to perform but may lack specificity and sensitivity compared to HPLC or LC‐MS. Capillary electrophoresis (CE) separates ionized species based on their size‐to‐charge ratio within a capillary tube. While CE requires minimal sample preparation and volume, it is relatively less common for metformin detection [27]. Table 1 summarizes various detection methods for metformin, highlighting their descriptions, advantages, and limitations across different sample types such as biological fluids, food products, and environmental samples.

**TABLE 1:** Conventional methods in detection of metformin.

Method	Description	Advantages	Limitations	Ref
HPLC	Utilizes a liquid chromatography technique to separate and quantify metformin.	Highly accurate and reliable. Can analyze multiple samples simultaneously.	Requires expensive equipment and expertise.	[23]
LC‐MS	Combines liquid chromatography with mass spectrometry for precise quantification and identification.	Very sensitive and capable of detecting low concentrations.	High cost and technical complexity.	[24]
GC	Involves vaporizing a sample and separating it in a gas phase.	Good for volatile components.	Not suitable for nonvolatile compounds like metformin without derivatization.	[25]
ELISA	A biochemical assay that utilizes antibodies to detect the presence of metformin.	Relatively quick and easy to perform.	May lack specificity and sensitivity compared to HPLC or LC‐MS.	[26]
CE	Separates ionized species by their size‐to‐charge ratio in a capillary tube.	Requires minimal sample preparation and volume.	Relatively less common for metformin detection.	[27]

Biosensors have revolutionized the field of detection by integrating biological sensing elements with transducer technologies to convert biological interactions into quantifiable signals [28, 29]. These devices are particularly advantageous in monitoring pharmaceutical compounds such as metformin due to their high specificity and sensitivity [28, 29]. Most recently, metformin biosensors have gained traction, with various structures being developed to optimize performance. These biosensors typically incorporate nanomaterials such as graphene, CNTs, and metal nanoparticles (NPs), greatly enhancing their electrochemical properties. For instance, graphene‐based biosensors exhibit exceptional conductivity and a high surface area, allowing for more active sites for metformin interaction, leading to improved sensitivity. The working principle of these biosensors often relies on electrochemical detection methods. When metformin interacts with the sensing element, it induces a change in current, potential, or impedance, which is then measured and correlated to the concentration of metformin present in the sample. This sensitivity is further enhanced through the use of nanomaterials, which facilitate faster electron transfer and amplify the electrochemical signals. Comprehensive studies on these biosensors investigate their fabrication processes, response times, detection limits, and overall performance in real‐world applications. The integration of innovative nanomaterials into biosensor technology not only enhances their functionality but also opens new avenues for the efficient detection of metformin across various domains, including healthcare, environmental monitoring, and food safety. A comprehensive examination of these aspects, including biosensor structures, the types of nanomaterials employed, and their operational principles, will be discussed in the following sections of this article, highlighting the innovations and efficiencies that modern biosensors bring to metformin detection.

## 4. Biosensor Methodology

**FIGURE 3:**
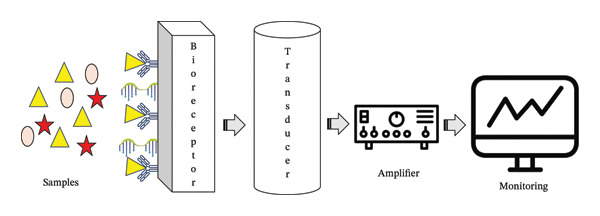
Schematic illustration of the biosensor structure.

Biosensors are analytical devices that integrate a biological recognition element with a physicochemical transducer to measure changes in biological processes and convert them into a quantifiable electrical signal, typically current or voltage [30, 31]. The biological recognition element can include enzymes, tissues, microorganisms, cells, nucleic acids, antibodies, or other biomolecules [29, 30]. The choice of biorecognition element, along with the enzyme or material properties, determines the transducer output and overall sensor performance [32]. The essential components of a biosensor are the analyte (the target substance being measured, e.g., glucose or bacteria), the bioreceptor (which specifically binds or reacts with the analyte), the transducer (which converts the biochemical interaction into an electrical signal), the electronics (for signal amplification, filtering, and processing), and the reader/display (for giving results in a user‐friendly format); see Figure 3 [33]. The reader is often custom‐designed according to the biosensor’s operational principle and represents a significant portion of the development cost.

Biosensors have found broad applications in waste monitoring, medical diagnostics, psychiatric condition management, health monitoring, agricultural research, forensic analysis, water quality assessment, environmental monitoring, and food safety [33]. In clinical settings, they are especially valuable for disease diagnosis, continuous patient monitoring, and chronic disease management. A prominent example is glucose biosensors, which are indispensable for diabetes management by enabling real‐time blood glucose tracking and helping patients maintain target levels [34]. These devices accelerate diagnosis, improve treatment decisions, and enhance overall patient outcomes.

A critical feature of electrochemical biosensors, the most extensively used type for many analytes, is the choice of biorecognition element, which mainly governs specificity and sensitivity. The physicochemical properties of the bioreceptor also influence the design of the transduction layer. Recent advances have introduced diverse biorecognition approaches for electrochemical lactate biosensors (and analogous systems), including metal oxides, enzymes, NPs, metal‐organic frameworks, and molecularly imprinted polymers.

Electrochemical biosensors stand at the forefront of modern analytical technology, offering rapid, sensitive, and selective detection of a wide range of biological and chemical substances. Their progress has profoundly influenced diagnostics, environmental monitoring, and food safety. A comparison of main biosensor types is provided in Table 2.

**TABLE 2:** Comparison of different types of biosensors.

Type of sensor	Working principle	Advantages	Disadvantages	Ref
Electrochemical	Measure current, voltage, or impedance changes due to biochemical reactions.	‐ High sensitivity ‐ Quick response time ‐ Small size	‐ May require careful calibration‐ May be affected by interfering substances	[34, 35]

Optical	Use light absorption, fluorescence, or reflectance to detect biological interactions.	‐ Label‐free detection ‐ High specificity ‐ Can be multiplexed	‐ Often more expensive‐ Limited in some biological environments	[36, 37]

Mass‐Based	Detect changes in mass due to biomolecular interactions, typically using quartz crystals.	‐ Real‐time monitoring‐ High sensitivity to mass changes	‐ Requires rigid setups‐ Limited to solid‐phase interactions	[38, 39]
Thermal	Measure temperature changes from biochemical reactions or cellular metabolism.	‐ Simple and cost‐effective‐ No labeling needed	‐ Lower sensitivity compared to electrochemical sensors	[40, 41]
Piezoelectric	Utilize changes in electrical charge in response to mechanical stress from binding events.	‐ High sensitivity‐ Real‐time detection	‐ Can be sensitive to environmental changes‐ Complexity in setup	[31, 42]

While electrochemical biosensors shine in sensitivity and response time, they can be vulnerable to interfering substances and often require careful calibration. Other types provide complementary strengths (e.g., label‐free operation in optical sensors or simplicity in thermal sensors), so the optimal choice depends on the required sensitivity, speed, cost, and operating environment.

Due to the limitations of conventional metformin detection methods (e.g., expensive instrumentation, time‐consuming sample preparation, or insufficient sensitivity/selectivity in complex matrices), there has been a marked shift toward nanomaterial‐based biosensors. Over the past decade, these advanced electrochemical biosensors have arisen as fast, sensitive, and selective alternatives. The following sections introduce the latest developments in metformin biosensors, highlighting innovations in design, nanomaterials, and transduction strategies, together with their emerging applications in healthcare and TDM.

## 5. Metformin Biosensors

**FIGURE 4:**
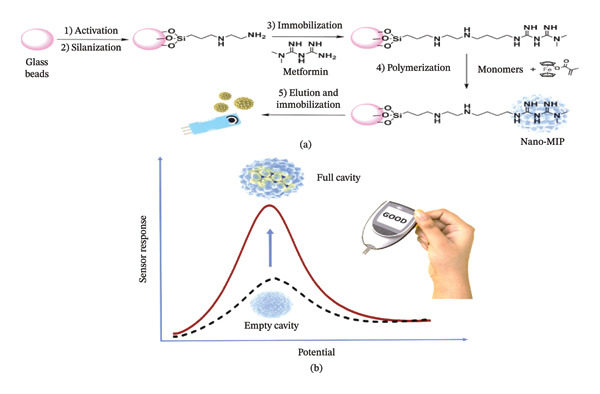
(a) Synthesis of metformin‐imprinted nanoactuators and (b) illustration of the sensor response during voltammetric analysis. The electrochemical response is depicted by plotting potential against current. The black dotted curve illustrates the nanoMIP with an empty cavity, while the red curve above it represents the nanoparticle with a filled cavity, indicating successful analyte recognition [46].

The study highlights the dual functionality of rare earth metal‐doped ZnO NPs (Sm‐ZnO, Dy‐ZnO, and Nd‐ZnO) for electrochemical sensing and photocatalytic applications. An eco‐friendly method was employed for their green synthesis using flower petals from *Rhododendron arboreum*. Comprehensive investigations into their structural, morphological, and optical properties were conducted using techniques such as XRD, XPS, and UV–visible spectroscopy. The XPS analysis confirmed the successful incorporation of +3 oxidation state dopants into the NPs. Additionally, these NPs exhibited high sensitivity in electrochemical sensing, for metformin hydrochloride detection in aqueous media [43]. A new electrochemical sensor (P‐g‐C_3_N_4_/MOF‐199/CPE) has been developed to measure metformin concentrations in pharmaceutical samples. In this sensor, the copper units in the MOF‐199 composite electrode specifically capture metformin molecules, significantly enhancing sensing selectivity. Additionally, phosphorus‐doped graphitic carbon nitrides (P‐g‐C_3_N_4_) further improve the sensor’s electrical conductivity and sensitivity [44]. An electrochemical biosensor for metformin (MET) has been created using a boron‐doped diamond (BDD) electrode modified with Nafion, focusing on how pH levels in electrolytes influence its properties. The performance of the modified electrode is evaluated using electrochemical impedance spectroscopy (EIS), cyclic voltammetry (CV), and atomic force microscopy (AFM). Results indicate that the Nafion‐modified BDD electrode shows significantly enhanced electrocatalytic activity for metformin oxidation compared to the unmodified BDD electrode [45]. Smart artificial nanoactuators were created using electroactive acrylamide polymer NPs through molecular imprinting. These NPs act as receptors that emulate the specificity of antibodies, effectively substituting enzymes in conventional biosensors and enabling highly sensitive and selective monitoring. The synthesis of electroactive polymer NPs involves incorporating ferrocene, which provides signaling capabilities. These actuators function by a swelling mechanism triggered by a specific electrochemical stimulus. Molecular recognition relies on molecular imprinting and the binding of the target molecule. The detection of metformin was accomplished using DPV across a broad concentration range, demonstrating a favorable limit of detection in plasma; see Figure 4 [46].

**FIGURE 5:**
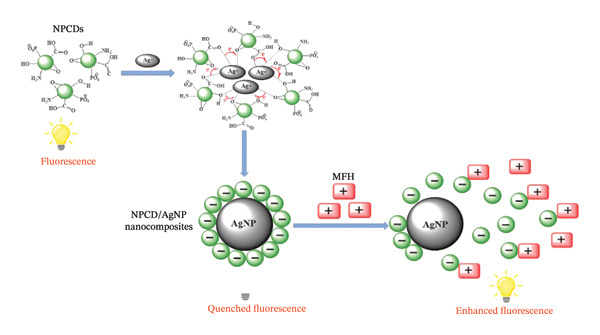
Diagram of the NPCD/AgNP nanocomposite‐based system used for MFH detection [53].

A new ratiometric fluorometric sensor has been created using dual‐emission nitrogen and sulfur codoped carbon dots (NS‐CDs) for the sensitive detection of metformin. The sensing mechanism is based on a distinctive disaggregation phenomenon that occurs under acidic conditions; the electrostatic repulsion between positively charged NS‐CDs and metformin molecules results in increased fluorescence emission [47]. The study presents a comparison of the sensitivity and selectivity between two modified wire membrane sensors enhanced with Al_2_O_3_ and NiO NPs and conventional wire membranes for quantifying the antidiabetic drug metformin hydrochloride (MTF) [48]. The Mg_12_O_12_ nanocluster has been modified with transition metals, specifically nickel (Ni) and zinc (Zn), to enhance the adsorption of metformin. The incorporation of Ni and Zn onto the nanocluster facilitates two distinct geometries, and the adsorption of metformin similarly results in two different geometries [49]. A sensitive electrochemical method, enhanced by multivariate optimization techniques, is introduced for measuring the antidiabetic drug metformin (MET) in pharmaceutical and serum samples. The sensor features a modified carbon paste electrode (CPE) using a copper–graphene nanocomposite (Cu‐G/CPE). This Cu‐G/CPE demonstrates excellent reproducibility and stability, high recovery rates, and benefits from being low‐cost, producing a low background current, and allowing for easy surface renewal [50]. In one study, an electrochemical sensor utilizing nitrogen‐doped CNTs (NCNTs) was developed for the ultrasensitive detection of metformin (MET) in the presence of Cu (II) ions. The remarkable conductivity and abundant π‐conjugated structure of NCNT enhance the electron transfer rate of the sensor and improve the adsorption of cation ions [51]. A FRET‐based fluorescent nanosensor, known as the Tb–phen–AgNP system, was developed for the sensitive detection of MET in tablet and serum samples. This approach leverages the enhancing effect of MET on the emission intensity of the Tb–phen complex, which is initially quenched by AgNPs through an energy transfer mechanism (turn off–on mode). Under optimal conditions, a strong linear correlation was found between MET concentration and the increased emission intensity of the Tb–phen–AgNP system, within the acceptable linear range [52]. One study introduced a fluorescence sensor utilizing NPCD/AgNP nanocomposites for the detection of metformin hydrochloride (MFH). Researchers initially harnessed the reducing properties of NPCDs to synthesize AgNPs from Ag + ions, which were then used to create the NPCD/AgNP nanocomposites; see Figure 5 [53].

**FIGURE 6:**
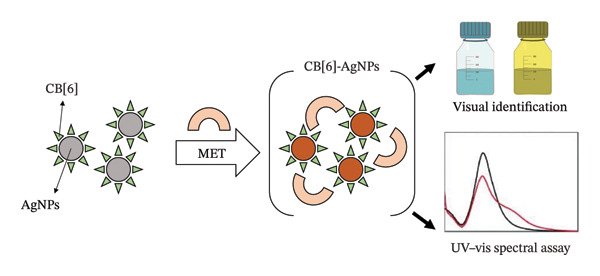
A schematic representation of the sensing process for the metformin (MET)‐induced aggregation of cucurbit[6]uril (CB [6])‐modified silver nanoparticles (AgNPs), adapted from Ref. [57].

An electrochemical sensor utilizing a CPE modified with a γ‐Fe_2_O_3_@hydroxyapatite/Cu(II) nanocomposite [γ‐Fe_2_O_3_@HAp/Cu(II)] was advanced for the determination of metformin (MET). The synthesized nanocomposite underwent thorough characterization using EDX, TEM, FTIR, SEM, and VSM techniques. The electrocatalytic oxidation of MET on the modified CPE was examined through CV and adsorptive stripping differential pulse voltammetry (ASDPV) [54]. A novel electrochemical biosensor based on a CPE modified with bacterial nanocellulose, copper oxide, and AgNPs (Ag NPs/Cu_2_O/CuO/BNC/CPE) was developed for the high‐sensitivity determination of metformin (MET). The morphology and structure of this bionanocomposite were characterized using FE‐SEM, EDS, ATR‐IR, mapping, DRS, and XRD techniques. In comparison to the standard CPE, the modified electrode exhibited significantly enhanced electrocatalytic activity for the oxidation of MET [55]. A highly sensitive sensor for the electrochemical determination of the antidiabetic drug linagliptin (LG) was developed using a CPE modified with iron oxide NPs (Fe_2_O_3_NPs). The electrochemical performance of LG was analyzed, and several dynamics were explored for the first time. This study reveals that the oxidation reaction of LG on the CPE/Fe_2_O_3_NPs is a one‐electron and one‐proton process, influenced by both diffusion and adsorption. Additionally, the simultaneous determination of LG with glucose and metformin (MET) was investigated [56]. A straightforward and sensitive assay for metformin (MET) that can be observed with the naked eye has been developed using cucurbit[6]uril (CB [6])‐modified AgNPs for the first time. The molecular interaction between CB [6] and MET is initially established, and the underlying recognition mechanism is further explored; see Figure 6 [57].

A straightforward and environmentally friendly fluorescence detection system has been established for the first time, utilizing the quenching effect of Cu(II) ions on the fluorescence of graphitic carbon nitride nanosheets (g‐C_3_N_4_ NS). This system records changes in restored fluorescence intensity upon the addition of metformin (MET). The g‐C_3_N_4_ NS were synthesized through the high‐temperature polymerization of melamine, followed by ultrasonication‐assisted liquid exfoliation [58]. A novel, cost‐effective, selective, and robust sensor has been created for the electrochemical detection of metformin (MET) using anodic stripping voltammetry. This sensor incorporates a nanosized mesoporous silica material functionalized with copper ions (SBA‐15‐Cu(II)) into the CPE. The sensor’s sensitivity was enhanced by increasing the accumulation of MET on the electrode surface through its complexation with copper ions [59]. A simple colorimetric method for determining metformin in drug formulations was conducted using environmentally friendly gamma‐cyclodextrin (γ‐CD) stabilized AgNPs (CD‐AgNPs). γ‐CD served as both the reducing agent for the metal salt and the stabilizing agent for the NPs [60]. Utilizing the host–guest molecular recognition ability of cucurbit [6]uril (CB [6]) modified on a gold surface, sensitive spectrophotometric and electrochemical techniques have been developed for detecting MET. Initially, CB[6]‐modified (AuNPs/CB[6]) were synthesized and subsequently characterized using UV–vis spectroscopy and TEM. The aggregation of AuNPs/CB[6] induced by MET resulted in color changes and alterations in the absorption spectrum, facilitating both visual detection and spectrophotometric quantification of MET [61]. A novel, sensitive, and straightforward method for detecting metformin has been reported. In this context, nanolayered manganese‐calcium oxide (NL‐MnCaO_2_) was synthesized and characterized using SEM, FTIR spectroscopy, and XRD techniques. The findings revealed that metformin inhibited the peroxidase‐mimicking activity of NL‐MnCaO_2_, and this inhibition intensified with increasing concentrations of metformin [62]. A selective and sensitive electrochemical sensor for detecting MET has been developed. This sensor was created using a composite of molecularly imprinted polymer, nickel cobaltite nanorods, and graphene oxide (MIP/NiCo_2_O_4_ NRs/GO) to modify a carbon cloth (CC) electrode. The sensor utilized the molecularly imprinted polymer for the specific recognition of MET, while the NiCo_2_O_4_ NRs/GO nanocomposite in the CC substrate enhanced the signal amplification [63]. A straightforward hydrothermal method was employed to transform expired metformin into valuable fluorescent carbon quantum dots (m‐CQDs) with sizes under 10 nm. The m‐CQDs exhibited a blue emission at 420 nm when excited at 350 nm. Notably, upon the addition of tetracycline (TC), the blue emission decreased, giving way to a greenish‐yellow emission at 530 nm [64]. Additionally, one study reports the eco‐friendly green synthesis of samarium‐doped zinc oxide (Sm‐doped ZnO) NPs using *R. arboreum* flower petal extract, eliminating toxic chemicals in line with sustainable nanotechnology. Structural, morphological, and optical properties were characterized by XRD, FESEM, HRTEM, UV–vis, and fluorescence spectroscopy. These platforms also showed excellent sensing performance for the antidiabetic drug metformin hydrochloride, highlighting their dual potential for environmental wastewater remediation and drug quality monitoring in pharmaceutical applications [65]. One research work investigates cerium oxide (CeO_2_) and iron‐doped cerium oxide (Fe–CeO_2_) NPs synthesized by a cost‐effective solution‐combustion method, with detailed characterization (TEM, SEM, XRD, BET, FTIR, UV–vis) confirming successful Fe incorporation, reduced crystallinity, altered morphology, and a narrowed optical bandgap. Fe‐doping significantly enhances photocatalytic degradation of textile dyes and improves electrochemical sensing performance for the antidiabetic drug metformin hydrochloride, achieving a higher electroactive surface area and acceptable LOD compared to pristine CeO_2_ [66]. More details of metformin biosensors are presented in Table 3.

**TABLE 3:** Metformin biosensors.

Type	Technique	NPs	Electrode	Sample	Linear range	LOD/sensitivity	Ref
EL	Spectroscopy	Nd‐ZnO	NA	Aqueous	NA	0.338 mA/Mcm^2^	[43]
EL	CVs, DPVs	P‐g‐C_3_N_4_/MOF	CPE	Pharmaceutical	0.5–1200 nM	0.15 nM	[44]
EL	SWVs, CVs, EIS	NA	BBD	Urine	NA	14 nM	[45]
EL	ATR‐FTIR spectrometer/MIP	Polymer	SPPE	Plasma	100 and 2000 pM	9 pM	[46]
EL	Fluorescence	NS‐CDs	NA	Biological	0.05–0.9 µM	15 nM	[47]
EL	FTIR spectra	Al_2_O_3_/NiO	NA	NA	1.0 × 10^−10^–1.0 × 10^−2^ mol L^−1^	5.0 × 10^−11^ M	[48]
EL	Quantum chemical	Mg_12_O_12_	NA	Biological	NA	NA	[49]
EL	CVs, DPVs	Cu‐G/CPE	CPE	Serum	10.4–1125.0 µM	3.4 µM	[50]
EL	NA	NCNT	NA	Aqueous	0.3–10 μmol L^−1^	9.6 nmol L^−1^	[51]
Optical	FRET‐based fluorescent	AuNPs	NA	Serum	(0.75–3.7) × 10^−6^ M	0.43 × 10^−6^ M	[52]
Optical	FRET‐based fluorescent	NPCD/AgNP	NA	Serum	2–100 μg/L	1.76 μg/L	[53]
EL	CVs	γ‐Fe_2_O_3_ @HAp/Cu(II)	CPE	Pharmaceutical/urine	0.1–80 μM	14 nM	[54]
EL	CVs	Cu_2_O/CuO/BNC	CPE	Tablets	0.1–76 and 76–1000.0 μM	42.3 nM	[55]
EL	CVs/SWV	Fe_2_O_3_NPs	CPE	Tablets/urine	0.03–86 μg/mL	8.0 ng/mL	[56]
EL/optical	UV–vis spectral	AgNO_3_	NA	Urine	3–750 μM	1 μM	[57]
EL/optical	Fluorescent	g‐C_3_N_4_ NS	NA	Biological	0.01–20 μM	3 nM	[58]
EL	DPV	SBA‐15‐Cu(II)	CPE	Pharmaceutical and biological	0.1–65 μM	30 nM	[59]
EL/optical	FTIR, UV–vis spectroscopy	CD‐AgNPs	NA	Pharmaceutical	0.2–1.25 µM	42 nM	[60]
EL/optical	UV–vis spectrometry/EIS	AuNPs/CB [6]	Gold	Pharmaceutical	6–700 μmol/L	1.35 pmol/L	[61]
EL/optical	Fluorometric	NL‐MnCaO_2_	NA	Biological	0.07–0.77 mM	0.17 μM	[62]
EL	EMIP	NiCo_2_O_4_ NRs/GO	CC	Serum/plasma	0.01–1.0 μM	0.947 nM	[63]
EL/optical	UV–vis spectrometry/EIS	*m*‐CQDs	NA	Pharmaceutical	NA	NA	[64]
Optical	CVs/UV–vis	Sm‐doped ZnO	NA	Water	NA	NA	[65]
EL/optical	XRD, BET, FTIR	Fe–CeO_2_	NA	Pharmaceutical	NA	6.65 mM	[66]

Abbreviation: NA, not applicable/not available.

Table 3 presents a comprehensive overview of various metformin biosensors, highlighting different types, techniques, and characteristics. The type categorized as EL includes various methods such as spectroscopy, CVs, differential pulse voltammetry (DPVs), and square wave voltammetry (SWVs). Notable materials used for NP development include Nd‐ZnO, P‐g‐C_3_N_4_/MOF, and Cu‐G/CPE, with electrodes ranging from CPE to screen‐printed electrodes (SPPE). Samples analyzed vary widely, including aqueous solutions, pharmaceuticals, urine, plasma, and biological samples. Linear detection ranges for these biosensors cover a broad spectrum, such as 0.5–1200 nM for P‐g‐C3N4/MOF and even extending from 0.1 to 65 μM for SBA‐15‐Cu(II). The LOD or sensitivity is notably impressive for several biosensors, with values such as 9 pM for polymer‐based sensors and 0.15 nM for P‐g‐C_3_N_4_/MOF. Optical techniques also feature prominently, utilizing fluorescence and FRET‐based approaches with materials such as AuNPs and AgNPs. These optical biosensors showcase a range of detection sensitivities, with LOD values such as 0.43 × 10−6 M for AuNPs in serum and 1.76 μg/L for NPCD/AgNP. Further advancements in electrochemical methods are represented in sensors employing materials such as γ‐Fe_2_O_3_ @HAp/Cu(II) and Cu_2_O/CuO/BNC, reflecting versatility in detecting Metformin concentrations in different matrices, including tablets and urine, with linear ranges from 0.1 to 80 μM and LODs as low as 14 nM. Additionally, the table lists biosensors that incorporate multi‐technique approaches, combining electrochemical and optical methods, showcasing the diverse strategies available for the sensitive detection of Metformin. Overall, the range of techniques, NP types, and sample matrices underline the advancements in biosensing technologies for Metformin, ensuring high sensitivity and specificity across various applications.

## 6. Common Bottlenecks Across Techniques

Most biosensors in Table 3 rely on nanomaterials (e.g., metal oxides such as Nd‐ZnO, Cu‐based composites, Fe_2_O_3_NPs, carbon nanomaterials such as g‐C_3_N_4_, graphene, CNTs, or quantum dots) to improve electrocatalytic activity or signal transduction. On the other hand, several recurring issues hinder performance: (A) Stability of nanomaterials: Long‐term operational and storage stability remains a main alarm, particularly for enzyme‐free electrochemical sensors using metal oxides or carbon‐based modifiers. Nanomaterials can suffer from leaching, aggregation, or surface passivation over time, leading to signal drift and reduced reproducibility in repetitive use or real‐sample matrices. For instance, while many reported sensors demonstrate good short‐term reproducibility, few studies rigorously assess stability beyond days or under changing environmental conditions (pH, temperature, humidity), which is critical for POC devices. (B) Anti‐interference performance: Biological and pharmaceutical samples (e.g., urine, serum, plasma, tablets) contain interferents such as ascorbic acid, uric acid, dopamine, glucose, and other electroactive species. Electrochemical sensors (the dominant type in Table 3) often face cross‐reactivity during direct oxidation of Metformin, especially at high potentials. Optical approaches (e.g., FRET‐based with AuNPs or fluorescence with NS‐CDs may experience background fluorescence or quenching from matrix components, though they generally offer better selectivity when using specific recognition elements such as MIPs. Multi‐technique hybrids (EL/Optical) show promise in mitigating interference but add complexity. (C) Cost and scalability for mass production: Many sensors incorporate expensive or synthetically complex nanomaterials (e.g., MOFs, rare‐earth doped oxides, or noble metal NPs such as Au/Ag). CPEs and screen‐printed electrodes (SPPE) are low‐cost and amenable to mass production, but integration of advanced modifiers increases fabrication steps and costs. Optical sensors often require sophisticated instrumentation (e.g., fluorometers or spectrometers), limiting portability and affordability compared to simple electrochemical setups. (D) Real‐sample applicability and matrix effects: While many sensors report detection in urine, serum, or pharmaceuticals, recovery rates and matrix effects are inconsistently addressed. Dilution or pretreatment is frequently needed, reducing practicality for direct analysis.

## 7. Horizontal Comparison and Priority Ranking of Technical Routes

Electrochemical sensors dominate Table 3 (∼80% of entries), leveraging techniques such as CV, DPV, SWV, and EIS for direct, label‐free detection with advantages in simplicity, rapid response, miniaturization, and low cost. They realize sub‐nM to μM LODs and broad linear ranges, making them suitable for POC and clinical monitoring. Conversely, they are prone to interference and electrode fouling in complex matrices.

Optical sensors (including FRET, fluorescence, UV‐Vis, and FTIR‐based) offer intrinsic advantages in selectivity (especially with specific probes) and visual/qualitative readout potential, but suffer from lower sensitivity in some cases, higher susceptibility to optical interference (e.g., turbidity), need for exterior light sources/detectors, and generally poorer portability.

Hybrid EL/Optical methods combine strengths (e.g., dual confirmation of signals) but increase complexity and cost.

For Metformin detection applications, the following priority ranking reflects key factors including sensitivity, selectivity, cost, stability, and practicality.

Electrochemical sensors that employ carbon‐based materials or non‐precious metal modifiers, such as Cu‐G/CPE, γ‐Fe_2_O_3_@HAp/Cu(II), and Cu_2_O/CuO/BNC, receive the maximum priority for most applications. They offer an excellent balance of low limits of detection (typically in the nM range), wide linear ranges, use of low‐cost CPE, effective anti‐interference properties provided by the modifiers, and strong potential for mass production and portable devices. These designs outperform noble‐metal‐based alternatives in cost‐effectiveness without sacrificing analytical performance.

Next are advanced electrochemical sensors incorporating MIP or sophisticated nanocomposite structures, for example EMIP with NiCo_2_O_4_ NRs/GO and P‐g‐C_3_N_4_/MOF. These provide superior selectivity in real samples and achieve ultra‐low LODs (sub‐nM levels), making them particularly suitable for clinical analysis of plasma or serum. However, long‐term stability and scalability for practical deployment still require more validation.

Optical sensors, including FRET‐based or fluorescence‐based systems such as those using AuNPs, NPCD/AgNP, or g‐C_3_N_4_ NS, rank lower. They are valued in scenarios where high selectivity is more important than rapid response or portability, such as laboratory‐based pharmaceutical quality control. Their lower ranking stems primarily from the need for specialized instrumentation and greater susceptibility to matrix‐related optical interference.

Hybrid approaches or those heavily reliant on spectroscopy, such as ATR‐FTIR combined with MIP or multi‐spectral UV‐Vis/EIS methods, hold niche value for confirmatory or reference testing but are assigned the lowest priority for routine or point‐of‐care use because of their complexity and limited suitability for field applications.

Overall, electrochemical strategies currently demonstrate the highest versatility and practicality for Metformin biosensing. To advance the field toward commercial point‐of‐care devices, future efforts should focus on enhancing nanomaterial stability (for example through protective coatings), improving anti‐interference capabilities (via selective membranes or machine learning‐assisted signal processing), and developing more cost‐effective and scalable fabrication processes.

## 8. AI‐Integrated Biosensors for Metformin Detection: Current Status and Research Gaps

The integration of artificial intelligence (AI) and machine learning (ML) with biosensors represents a rapidly evolving frontier in analytical and personalized medicine [67]. AI/ML techniques such as convolutional neural networks (CNNs), long short‐term memory networks (LSTMs), support vector machines (SVMs), and reinforcement learning have demonstrated substantial potential in enhancing biosensor performance across various analytes [68, 69]. These methods excel at real‐time signal denoising, feature extraction from complex electrochemical or optical data, interference mitigation, calibration‐free operation, multimodal data fusion, and predictive modeling of biomarker trends [68, 69]. In diabetes management, AI has been successfully applied to continuous glucose monitoring (CGM) systems, enabling glycemic prediction, personalized insulin dosing recommendations, and closed‐loop “sense‐and‐act” platforms that integrate wearables with automated therapy adjustments [70, 71].

Despite these advances in AI and ML for biosensors, no dedicated AI‐based biosensors specifically for metformin detection or monitoring have been developed or published to date. Very few studies even explore this direction. [43, 72]. Wearable electrochemical and microneedle‐based platforms do exist. They achieve simultaneous in vivo monitoring of glucose and metformin in interstitial fluid (ISF). These use nanoenzyme‐functionalized sensors and DPV [73]. However, these systems rely mainly on traditional signal processing. They use chemometric approaches only. They lack embedded AI or ML. There is no advanced analysis, no noise‐adaptive calibration, no PK/PD prediction, and no intelligent decision support.

This represents a clear research gap. It limits progress in intelligent, personalized metformin TDM. Future work should address this limitation. Similarly, broader wearable biosensors for diabetes (e.g., sweat‐ or ISF‐based devices tracking glucose alongside other metabolites) increasingly incorporate ML for multimodal data interpretation and personalized insights, but metformin‐specific quantification remains unaddressed in an AI‐enhanced context.

This absence constitutes a major gap in biosensor technology, particularly for metformin—the most widely prescribed oral antidiabetic drug for Type 2 diabetes. Conventional metformin TDM is limited by infrequent blood sampling, high interpatient pharmacokinetic (PK) variability, and challenges in real‐time assessment of drug exposure in complex matrices.

## 9. Conclusion and Future Direction

The progresses in metformin biosensors represent a major progress forward in analytical chemistry, showcasing notable improvements in sensitivity, specificity, and applicability across complex matrices such as pharmaceutical formulations and biological fluids. The synergistic integration of electrochemical (including voltammetric and amperometric approaches) and optical (such as fluorescence or surface‐enhanced Raman scattering) detection strategies, often augmented by cutting‐edge nanomaterials such as graphene oxide, CNTs, MIPs, metal oxide nanorods (e.g., NiCo_2_O_4_), and copper‐modified multiwalled CNTs, has allowed ultrasensitive, selective, and rapid quantification of metformin, even at nanomolar levels in challenging real‐world samples.

These biosensors provide a consistent, cost‐effective platform for quality control in pharmaceuticals and TDM in clinical settings, addressing key needs in diabetes management where precise metformin dosing is critical to efficacy and minimizing side effects. Notably, emerging wearable and skin‐interfaced designs have revealed promising correlations between sweat and plasma metformin concentrations, opening pathways for noninvasive, real‐time PK and pharmacodynamic (PD) monitoring. This progress not only strengthens point‐of‐care capabilities but also holds substantial promise for personalized medicine, enabling better adherence and adjustment of antidiabetic therapy.

Looking ahead, numerous key directions warrant focused research to translate these laboratory successes into widespread clinical and commercial impact. First, further enhancement of selectivity and long‐term stability remains crucial, particularly in complex biofluids prone to interference; advanced nanomaterials combined with MIPs or aptamers could mitigate fouling and biofouling while extending sensor lifespan. Second, miniaturization and integration into lab‐on‐a‐chip, microneedle‐based, or fully wearable platforms would enable convenient, continuous point‐of‐care diagnostics and even closed‐loop systems that couple metformin detection with automated drug release or alerts for dose optimization.

Third, leveraging artificial intelligence and machine learning algorithms for advanced signal processing, pattern recognition, and predictive analytics could refine data interpretation from noisy sensor outputs, facilitating personalized treatment regimens based on individual PK/PD profiles. Fourth, expanding multiplexed detection capabilities to simultaneously monitor metformin alongside related analytes such as glucose, other antidiabetic agents, metabolic biomarkers, or emerging indicators such as metal ions would support holistic metabolic profiling for diabetes and comorbidities.

Finally, greater emphasis on wearable integration and clinical validation through large‐scale human trials is crucial to assess real‐world performance, biocompatibility, and regulatory compliance. Such developments could revolutionize diabetes care by enabling proactive, patient‐centered monitoring, reducing complications, and extending benefits to broader applications in metabolic health management. Overall, continued interdisciplinary efforts in materials science, sensor engineering, and clinical translation promise to elevate metformin biosensors from promising research tools to transformative healthcare solutions.

## Author Contributions

Sajjad Jafarzadeh: conceptualization, literature search, writing original draft (introduction, methodology, and figures/tables), visualization, and manuscript editing. Elham Shaterian: conceptualization, analysis of nanomaterial‐based sensors, writing on bottlenecks and technical ranking, critical revision, and final approval. Samira Jafarisis: literature review on wearable platforms, writing on clinical implications and translation challenges, and manuscript review. Mahya Mohammadi: data curation, preparation of Table 3, drafting advantages/limitations sections, and critical revision. Hossein Azizian: literature search on electrochemical mechanisms, writing on specific modifiers, and accuracy review. Shahla Shiri: conceptualization, supervision, writing on AI‐integrated biosensors and research gaps, critical revision of full manuscript, and corresponding authorship. Ahmad Mobed: conceptualization, project coordination, synthesis of biosensor innovations, writing outlook/future perspectives, critical revision, and corresponding authorship.

## Funding

The authors have nothing to report.

## Disclosure

All authors read and approved the final manuscript.

## Conflicts of Interest

The authors declare no conflicts of interest.

## Data Availability

The authors have nothing to report.
